# Development and Angiogenic Potential of Cell-Derived Microtissues Using Microcarrier-Template

**DOI:** 10.3390/biomedicines9030232

**Published:** 2021-02-25

**Authors:** Gerard Rubí-Sans, Irene Cano-Torres, Soledad Pérez-Amodio, Barbara Blanco-Fernandez, Miguel A. Mateos-Timoneda, Elisabeth Engel

**Affiliations:** 1 Biomaterials for Regenerative Therapies Group, Institute for Bioengineering of Catalonia (IBEC), The Barcelona Institute of Science and Technology (BIST), 08028 Barcelona, Spain; grubi@ibecbarcelona.eu (G.R.-S.); irene.cano89@gmail.com (I.C.-T.); sperez@ibecbarcelona.eu (S.P.-A.); bblanco@ibecbarcelona.eu (B.B.-F.); 2 Networking Research Center on Bioengineering, Biomaterials and Nanomedicine (CIBER-BBN), 28040 Madrid, Spain; 3 IMEM-BRT Group, Department of Material Science, Escola d’Enginyeria de Barcelona Est (EEBE), Technical University of Catalonia (UPC), 08019 Barcelona, Spain; 4 Bioengineering Institute of Technology, Department of Basic Science, Universitat Internacional de Catalunya (UIC), 08195 Barcelona, Spain

**Keywords:** poly-lactic acid microcarriers, Cultispher^®^ S, rat bone marrow mesenchymal stem cells, microtissue, cell-derived matrix, angiogenesis

## Abstract

Tissue engineering and regenerative medicine approaches use biomaterials in combination with cells to regenerate lost functions of tissues and organs to prevent organ transplantation. However, most of the current strategies fail in mimicking the tissue’s extracellular matrix properties. In order to mimic native tissue conditions, we developed cell-derived matrix (CDM) microtissues (MT). Our methodology uses poly-lactic acid (PLA) and Cultispher^®^ S microcarriers’ (MCs’) as scaffold templates, which are seeded with rat bone marrow mesenchymal stem cells (rBM-MSCs). The scaffold template allows cells to generate an extracellular matrix, which is then extracted for downstream use. The newly formed CDM provides cells with a complex physical (MT architecture) and biochemical (deposited ECM proteins) environment, also showing spontaneous angiogenic potential. Our results suggest that MTs generated from the combination of these two MCs (mixed MTs) are excellent candidates for tissue vascularization. Overall, this study provides a methodology for in-house fabrication of microtissues with angiogenic potential for downstream use in various tissue regenerative strategies.

## 1. Introduction

In order to successfully mimic native tissues ex-vivo to restore or replace injured ones, great efforts have been made towards the development of modular tissue engineering (TE), where microstructural functional units are assembled to create complex tissue constructs [1]. In contrast with traditional top-down strategies, scaffold modularity allows the generation of complex structures that recapitulate native tissue architecture [2,3,4,5]. Moreover, bottom-up strategies allow to mimic tissue heterogeneity by reproducing its cellular microenvironment and biochemical properties, and direct physical arrangement by assembling small building blocks into macroscale tissue-like constructs [5,6]. Currently, modular constructs produced by functional subunits include different strategies, such as cell-laden hydrogels [7,8], cell-sheets [9,10], spheroids [11,12,13], direct tissue printing [14], and cell-laden microcarriers (MCs) [15,16,17].

From these strategies, cell-laden MCs represent a promising option in bottom-up TE applications. Their fabrication methods, material, size, porosity, coating, or encapsulating capacity, among other features, make them versatile candidates for many regenerative/substitutive applications of different human tissues [6,18,19]. An example of commercially available MC is crosslinked gelatin Cultispher^®^ S, which has been used to induce bone regeneration [18,19,20,21,22]. Another promising alternative to decellularized tissues/organs is the in vitro production of cell-secreted ECM scaffolds, the so-called cell-derived extracellular matrix (CDM) [23,24]. CDMs recreate in vivo environments under controlled in vitro conditions, allowing the generation of functional microtissues (MTs) [25,26]. CDMs present customizable features when these are combined with bioactive biomaterials [24].

Herein, we present a methodology to produce 3D MTs by seeding rat bone marrow mesenchymal stem cells (rBM-MSCs) at the surface of polymeric MCs. This 3D modular architecture aims to induce CDM deposition that aggregates into a tissue-like construct. We compared poly-lactic acid (PLA) MCs, previously developed in our group (PLA MTs) [27] versus the commercially available Cultispher^®^ S gelatin MCs (Cultispher^®^ S MTs), as well as combining both particles (mixed MTs) to produce MTs. Since de novo angiogenesis and vascularization of any injured region is crucial for a successful tissue and function regeneration [28,29], MTs angiogenic potential was assessed using the Chick embryo Chorioallantoic Membrane (CAM) model. This novel strategy can be relevant to produce auto- and allografts for tissue engineering applications in tissues and organs that require an extensive vessel network formation, such as bone, dermis, or muscle. Moreover, MTs can be used for disease modeling, such as in cancer progression and metastasis events.

## 2. Materials and Methods

### 2.1. Materials

Poly(lactic acid) (Purasorb^®^ PLDL 7038; 3.8 dL/g viscosity; Mw≈ 850,00 Da) was obtained from Corbion (Amsterdam, The Netherlands). (-)-Ethyl-L-lactate (purity > 99.0%), ammonium hydroxide (NH_4_OH), polyvinyl alcohol (PVA, 9–10 kDa, 80% hydrolyzed), *N*-hydroxysuccinimide 98% (NHS), Triton^®^ X-100, Tris-EDTA 100X, and Sigmacote^®^ were acquired from Sigma-Aldrich (Madrid, Spain). Cultispher^®^ S gelatin microcarriers were kindly provided by Percell Biolytica (Astorp, Sweden). Nunc™ 96-Well Polystyrene Round Bottom Microwell Plates with non-treated surface were acquired from Thermo Fisher (Spain). Human recombinant collagen type I (hrCol I) was purchased from FibroGen (San Francisco, CA, USA). Sodium hydroxide (NaOH) was purchased from Panreac (Barcelona, Spain). 1-(3-Dimethylaminopropyl)-3-ethylcarbodiimide (EDAC), Glycine BioUltra, and Vectashield^®^ Antifade Mounting Medium were acquired from Acros Organics (Amsterdam, The Netherlands), Fluka (Zaragoza, Spain), and Vectorlabs (CA, USA), respectively.

### 2.2. Poly-Lactic Acid Microcarriers Fabrication, Biofunctionalization and Characterization

Poly-lactic acid microcarriers (PLA MCs) were prepared by an emulsion/solvent evaporation technique, using ethyl-lactate as solvent [27]. Briefly, a 3.5% *w*/*v* PLA solution in ethyl-lactate was extruded through a double-bore needle (inner 30G) at a dispensing rate of 10 mL/h. A nitrogen gas coaxial flow (outer 22G) at 1 atm was used for breaking the solution jet into droplets. PLA MCs were formed by precipitation into a hydroalcoholic coagulation bath (0.3% *w*/*v* PVA in 70% *v*/*v* ethanol). Finally, MCs were washed and sieved through a 300 and a 40 μm strainer to remove any large aggregate and smaller than 40 µm particles. MCs size and size distribution were assessed using a Leica E600 optical microscope and calculated employing FIJI (ImageJ, v. 1.53c) software [30].

PLA MCs’ surface modification was performed by covalently attaching human recombinant collagen type I (hrCol I) to foster cellular response and adhesion to the material [31,32]. First, PLA ester bonds were hydrolyzed with 0.5M NaOH for 10, 30, and 60 min. Then, exposed -COOH terminal groups were activated with 0.1M/0.2M EDC/NHS solution in 70% ethanol for two hours2 h. Activated MCs were incubated in hrCol I overnight (100 µg/mL in PBS). Finally, functionalized MCs were washed with water and freeze-dried. MCs were stored at 4 °C until used.

### 2.3. Cell Culture

rBM-MSCs were isolated from long bones of 2–4 weeks old Lewis rats by the experimental animal service of the Scientific Park of Barcelona (SEA-PCB). Rats were anesthetized with 5% isoflurane and sacrificed in a CO_2_ saturated atmosphere [33]. rBM-MSCs were cultured in aDMEM (Gibco, Barcelona, spain) supplemented with 10% FBS (Sigma, Madrid, Spain), 1% penicillin/streptomycin (100 µg/mL) and 1% L-glutamine (2 mM; Sigma). Passages between 4–6 were used in all experiments. All animal care protocols were approved by the Committee on Ethics and Animal Experiments of the Scientific Park of Barcelona (Permit No. 0006S/13393/2011, 2011).

### 2.4. Microcarrier Cell Seeding and Microtissue ProductionCell Seeding &Microtissue Production under Static Conditions

Three different culture formats were tested for MT formation under static conditions—(i) 96-well plates (U96; Nunc, U-shaped bottom non-treated surface #262162); (ii) 6 mm diameter and 5 mm depth wells in 1 cm^3^ polydimethylsiloxane (PDMS) molds; and (iii) ultra-low attachment 24-well plates (24w) tilted 45°,45° allowing the accumulation of cell-seeded MCs at the bottom of the wells. Standard protocols were initiated with 3 mg MCs per well. This was adapted for U-96 format reducing MCs content six times (0.5 mg). For each of these culture formats, three different cell/MCs seeding protocols were determined—(i) 25,000 cells/mg MCs, (ii) two-step seeding of 12,500 cells/mg MCs with an interval of 20 min, and (iii) 50,000 cells/mg MCs. Briefly, PLA MCs were rehydrated and sterilized in 70% *v*/*v* ethanol for 12 h prior to cell culture. Then, repeated washings were performed with sterile PBS until the culture medium was added. Cultispher^®^ S MCs were sterilized according to the manufacturer’s instructions. MCs were placed in the wells or molds, and rBM-MSCs cell suspension was added on top of the MCs. Cells were kept in a 37 °C, 5% CO_2_ humidified incubator. Cell medium was replaced every 2–3 days, for a total culture period of 21 days.

### 2.5. Microcarrier Cell Seeding and Microtissue ProductionCell Seeding & Microtissue Production under Dynamic Conditions

A 250 mL spinner flask device was used (BellCo, NJ, USA) for dynamic seeding. A hydrophobic layer of Sigmacote^®^ was created on the glass surface to avoid protein adsorption. Hydrated PLA MCs were placed inside autoclaved spinner flasks alongside 100 mL cell suspension in cell medium. Studied parameters under spinner flask dynamic seeding were: (I) The stirring regime, (II) serum content, (III) cell/MC ratio, and IV) seeding time. Two different stirring regimes were tested, 3 min 30 rpm/27 min 0 rpm and 15 min 30 rpm/15 min 0 rpm for 8 h. The spinner flask bioreactor was placed on a multiple magnetic stirrer block (Biosystem 4 Direct, Thermo Scientific, Barcelona, Spain) inside cell culture incubators (5% CO_2_, 37 °C) for 6 h. The effect of serum deprivation was also evaluated and compared with complete cell media (10% FBS). Different cell numbers per MC were tested (8, 10, or 12 cells per MC) to ensure maximum colonization. In addition, the optimal seeding time in the spinner flask was assessed (4, 8, 24, and 48 h). In all cases (II, III, and IV) an intermittent agitation (3 min 30 rpm/27 min 0 rpm) was used during the seeding. For 24 and 48 h cultures, intermittent agitation was only maintained during the first 8 h of incubation, and then continuous stirring was applied. Afterwards, 3 mg of cell-seeded MCs were transferred to each well of non-adherent 24-well plates. Plates were kept at 37 °C and 5% CO_2_ in a 45° titled position to promote the MT formation for 21 days.

Once determined the optimal culture conditions for MTs fabrication, PLA (PLA MT), Cultispher^®^ S (CultiS MT), and a combination of both PLA and Cultispher^®^ S (1:1) (mixed MT) MTs were produced following the established protocol for culture periods of 1, 7, 14, and 21 days.

### 2.6. Cell-Seeded Microcarriers’ Vital Staining

Cell viability after cell seeding in MCs surface was evaluated through life/dead staining using Calcein-AM and propidium iodide according to the manufacturer’s protocol (ThermoFisher). Samples were imaged under E600 Leica microscope (Wetzlar, Germany). In order to dismiss unspecific attachments, we introduced the concept of MC colonization rate (MCCR) as the number of MCs with three or more cells attached to them, divided by the total amount of MCs (Equation (1)).

(1) $$\mathrm{MCCR}=\frac{N{}^{\circ}\;\mathrm{of}\;\mathrm{MC}\;\mathrm{colonized}\;\mathrm{by}\;\mathrm{three}\;\mathrm{or}\;\mathrm{more}\;\mathrm{cells}\;}{\mathrm{Total}\;\mathrm{CR}\;\mathrm{number}}\;\times 100$$

### 2.7. Microtissue Size and Morphology Size & Morphology

MT size and morphology were evaluated using Leica stereomicroscope (Wetzlar, Germany), and ultra-high resolution field emission scanning electron microscopy (SEM, NOVA NanoSEM 230, FEI Company, Madrid, Spain). After 7, 14, and 21 days in culture, MTs were fixed in 4% PFA for 10 min at 4 °C, washed twice in cold PBS, and dehydrated in an increasing alcohol gradient. Then, samples were dried through critical point drying, and carbon sputtered. The size was analyzed by FIJI software (v. 1.53c) [30].

### 2.8. Cell Proliferation

Quant-IT Picogreen double-stranded DNA (dsDNA) assay kit (Invitrogen, Co Dublin, Ireland) was used to quantify total DNA to assess cell proliferation. MTs were collected at days 1, 7, 14, and 21, washed with PBS, and stored in tris-EDTA (TE) at −20 °C. MTs were homogenized by 15 s sonication in ice and using micro-tube adapted pestles. Three freeze-thaw cycles were performed (−80 °C/RT). Samples were centrifuged to remove MCs (5 min, 4000 g, 4 °C) and incubated with Quant-IT Picogreen reagent solution for 5 min at RT in the dark. Fluorescence was measured at 480/520 nm (excitation/emission) using a spectrophotometer plate reader (Infinite M200 PRO, Tecan, Barcelona, Spain).

### 2.9. Protein Deposition Quantification

BCATM Protein Assay kit (Pierce, Thermo Scientific) was used to quantify total protein from the MTs. Samples were collected at 1, 7, 14, 21 days and were homogenized in Mammalian Protein Extraction Reagent (M-PER) as described in Section 2.8. Three freeze-thaw cycles were performed (−80 °C/RT). Samples were centrifuged to remove MCs (5 min, 4000 g, 4 °C) and the supernatant was used for protein quantification according to the manufacturer’s protocol. Absorbance was measured at 562 nm using a spectrophotometer plate reader (Infinite M200 PRO plate reader, Tecan).

### 2.10. Immunofluorescence Staining

MTs ECM composition was assessed by immunofluorescence staining. MTs were collected after 21 days in culture and fixed in 4% PFA, dehydrated in increasing sucrose solutions (5%, 10%, and 30% *w*/*v*), and embedded in Tissue-Tek^®^ optimal cutting temperature (O.C.T.) Compound (Sakura^®^ Finetek, VWR, Barcelona, Spain). Samples were stored at −80 °C for 24 h. 25 µm thick sections were cut using Leica CM3050 S Research Cryostat.

Before staining, slides were thawed, and O.C.T was removed by MiliQ H_2_O washes. Cells were permeabilized (0.1% *v*/*v* Triton in 0.15% *w*/*v* glycine/PBS) for 10 min and blocked (1% *w*/*v* BSA and 10% goat serum in 0.15% *w*/*v* glycine/PBS) for 30 min. Then, slides were incubated with primary antibodies (1/500 in blocking solution, overnight at 4 °C, Table 1). After several washings, samples were stained with the secondary antibodies (1/1000) for one hour1 h at RT in dark conditions (Table 1). Then, the cell cytoskeleton and nuclei were stained with Phalloidin-Rhodamine (100 nM, 20 min, RT) and DAPI (1 µg/mL, 1 min, RT), respectively. Samples were mounted in Vectashield^®^ Antifade Mounting Medium and imaged by confocal microscopy (LSM780, Zeiss, Jena, Germany).

### 2.11. Microtissue Decellularization

MTs were decellularized by incubating with 1% Triton X-100 in a 0.1% NH_4_OH 30 min at 37 °C. Then, a DNase I treatment was later applied (30 µg/mL, 30 min, 37 °C).

### 2.12. Microtissue Angiogenic Potential—CAM Model

The angiogenic potential of the CDMs was assessed using the CAM model. Shell-less cultured ex-vivo models were adapted from a previously described protocol [34]. Fertilized chicken eggs were purchased from a local farm (Granja Gibert SA, Barcelona, Spain) and stored in a humidified incubator at 37 °C. After three days, the eggshell was aseptically and carefully cracked, transferring embryos into sterile Petri dishes (15 cm diameter). Embryos were kept in the incubator for six days. On the ninth day of development, 14-day PLA, mixed and Cultispher^®^ S decellularized MTs were prepared for implantation.

Implant preparation consisted of a single 6 mm circle nylon mesh (180 µm, Merck Millipore, Burlington, MA, USA) which guided implant CAM position over time. Decellularized MTs were carefully placed on top of nylon meshes, and they were embedded in 30 µL rat tail collagen type I solution (Opticol, Cell Guidance Systems, MO, US). As a negative control condition, polycaprolactone (PCL) irregular macroparticle (same macroscopic size as sample MTs) were used to simulate 3D microstructure. As a positive control condition, 200 ng vascular endothelial growth factor (VEGF) was added into collagen type I embedding solution covering PCL macroparticle implants. Implants were let to polymerize in a 37 °C incubator for 45 min. For the implantation, five implants per embryo were placed in areas containing fine vessels, avoiding any large blood vessels. Six to ten embryos per condition were used. After three more days of incubation, embryos were sacrificed by decapitation, and 10% formalin solution was used to fix the CAM for 30 min. Finally, scaffolds and their surrounding CAM tissue (1 cm around) were excised, and images were taken with an Olympus MVX10 Microscope. MTs’ angiogenic potential was quantitatively measured by determining vascular density in MTs. FIJI software was used following the script described in the supplementary information (Fiji macro S1).

### 2.13. Statistical Analysis

Results were statistically analyzed using GraphPad Prism 6 and expressed as mean and standard deviation of the replicates (*n* = 3, unless otherwise stated). One or Two-way Analysis of Variance (ANOVA) was used. Multiple comparisons test was performed using Tukey’s tests.

## 3. Results and Discussion

Decellularized in vitro CDMs is a promising methodology for developing biomimetic TE constructs [25,35,36,37]. The ECM secretion can be directly enhanced by the 3D architecture of scaffolds [38]. In this regard, MCs offer three-dimensional (3D) physical support for cells to sense and respond to complex architectures, while their biocompatibility and biodegradability allow for their in situ replacement by native tissues [27], making MCs suitable candidates for CDMs fabrication. In our study, we have determined the optimal conditions for the in vitro creation of CDM MTs from PLA, Cultispher^®^ S and mixed MCs.

### 3.1. PLA Microcarrier Characterization

**Figure 1:**
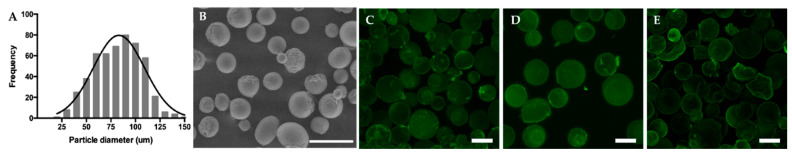
Poly-lactic acid (PLA) Microcarrier production and characterization. (**A**) Particle size distribution histogram. (**B**) PLA microcarrier (MC) SEM image, scale bar = 200 µm. (**C**–**E**) Collagen type I (green) staining of the functionalized MCs after 10, 30, and 60 min of 0.5 M NaOH hydrolysis, scale bars = 50 µm.

Using PLA/ethyl-lactate extrusion through a doubled-pore needle we obtained spherical MCs [27] with an average diameter of 81.85 ± 23.25 µm (Figure 1A). The MCs polydispersity adds structural heterogeneity to MTs, representing an advantage to better fit defects and adapt to injured sites. Moreover, this approach is inexpensive, non-toxic, and environmentally friendly. The MCs’ size and morphology were also assessed using SEM, confirming their size, polydispersity, and spherical shape (Figure 1B). MCs were surface-functionalized with collagen type I to improve their cell-adhesion properties. MCs were exposed to 10-, 30-, and 60-min hydrolysis, and collagen type I was covalently bound through ECD/NHS chemistry. Confocal images showed no qualitative differences between the different conditions tested (Figure 1C–E). Ten minutes hydrolysis was chosen to avoid polymer roughness and steric hindrance [31], to obtain uniformly functionalized spherical PLA MCs for downstream 3D cell culture.

### 3.2. Microtissue Development and Optimization

**Figure 2:**
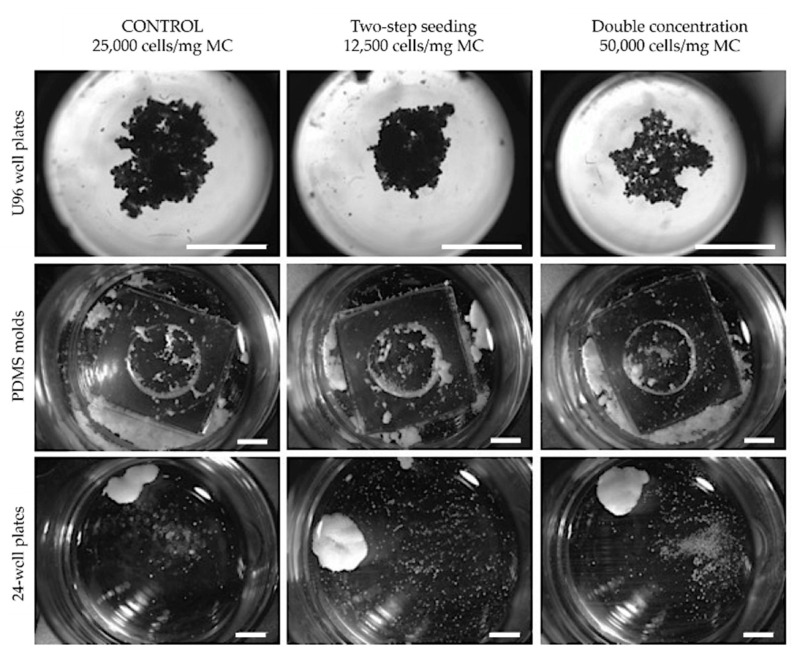
Microtissues (MT) fabrication and optimization under static conditions. Static cell-seeding in U96 well plates, polydimethylsiloxane (PDMS) molds, and 24-well plates at different cell/MC ratios, scale bars: U96 conditions = 3 mm; PDMS molds and 24 well plates = 2 mm.

MCs-assisted MT formation is based on the intrinsic capacity of cells to self-assembly during 3D culture through ECM deposition [39]. Static MTs formation was performed using different cell culture conditions and seeding densities. The cell culture conditions included the usage of various strategies, such as PDMS molds, U96 (u-shaped bottom surface), and flat 24-well low-attachment plates (Figure 2). In PDMS molds, no MTs were formed during 21 days of culture at any cell seeding condition, with cells spreading on PDMS and MCs randomly distributed in the wells. In U96 plates and 24-well plates cells were found attached to MCs and deposited ECM embedding them, forming MTs. No significant differences were found in the MTs size between the different cell-seeding conditions tested in U96 well plates. However, by using low-attachment surfaces that promote cell aggregation [11,40], differences in seeding protocol were observed. The two-step seeding protocol with a final cell/MC concentration of 25,000 cells/mg MC yielded statistically significant larger MTs (16.07 ± 2.83 mm^2^, *p* ≤ 0.001) than the two other conditions (8.71 ± 2.40 mm^2^ for 50,000 cells/mg; and 7.58 ± 1.11 mm^2^ for control condition) (Figure 2). Between U96 and 24-well plates approaches, MT size statistical differences were only found in the two-step seeding of 12,500 cells/mg MC (16.07 ± 2.83 mm^2^ for 24-well; 6.76 ± 2.12 mm^2^ for U96). MTs generation in 24-well plates resulted in the formation of dense structures easy to manipulate. Moreover, low-attachment plates also promoted cell attachment to PLA MCs. In contrast, U96 conditions showed a less dense structure where pores along the MT were easily observed (Figure 2).

**Figure A1:**
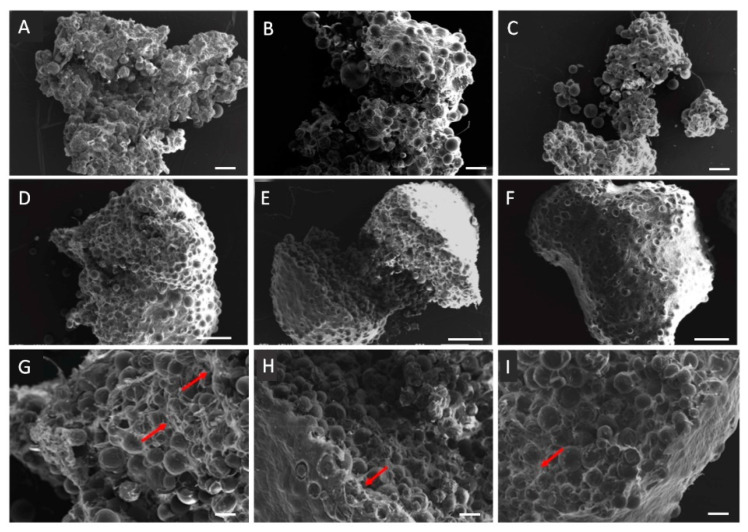
MT SEM images from (**A**–**C**) U96 well-plates with 25,000 cells/mg MC, 12,500 cells/mg MC two-step seeding and 50,000 cells/mg MC, scale bars = 200 µm; (**D**–**F**) 24 well-plates with 25,000 cells/mg MC, 12,500 cells/mg MC two-step seeding and 50,000 cells/mg MC, scale bars = 500 µm; and (**G**–**I**) transversal sections from 24 well-plates MTs with 25,000 cells/mg MC, 12,500 cells/mg MC two-step seeding and 50,000 cells/mg MC, scale bars = 100 µm. Arrowheads pointing fibrillar ECM.

Cell adhesion and ECM deposition were analyzed with SEM. ECM secretion following 21 days was not uniform between the different cell culture conditions. U96 MTs exhibited a non-complete CDM layer on the surface in both the two-step seeding and control condition (Figure A1A,B), and almost no CDM was found at 50,000 cell/mg condition (Figure A1C). In line with previous observations [41,42], we found that as cell proliferation and ECM deposition occurs, MTs contract from half-moon shape to spheres.

In contrast, the use of 24-well plates revealed an abundant fibrous, dense, and uniform CDM secreted by cells on top of MCs (Figure A1D–F). Transversal sections of MTs in all 24-well plates exposed a thin fibrillar network in between MCs (Figure A1G–I). Qualitative differences can be observed between MTs’ surface and core, where cell density and the amount of CDM were significantly lower than in the surface. Therefore, a 24-well plate culture strategy was used for the consequent experiments.

**Figure 3:**
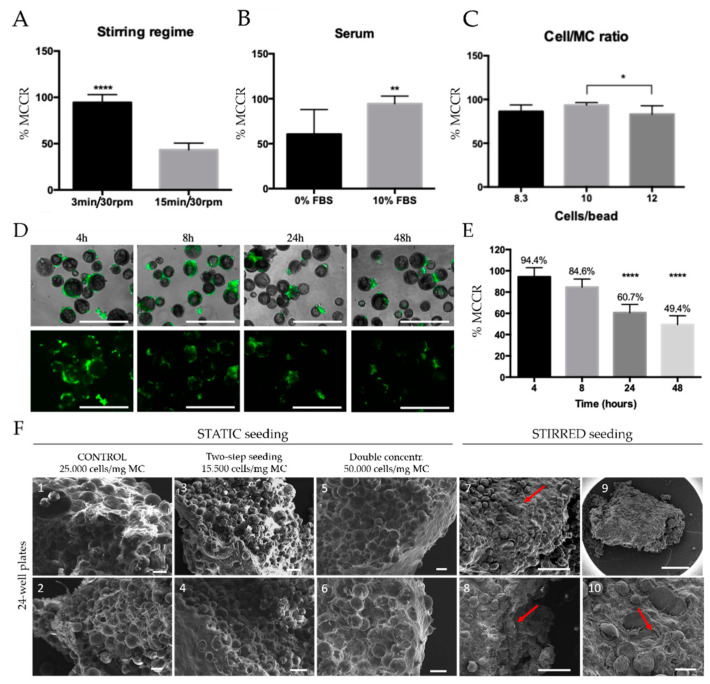
MT fabrication and optimization under dynamic conditions. (**A**) Dynamic MC colonization, stirring regime evaluation, scale bars = 500 µm, (**B**) serum content, scale bars = 500 µm, (**C**) cell/MC ratio, scale bars = 500 µm, and (**D**) cell seeding time on MCs, scale bars = 500 µm, alive cells in green, dead cells in red. (**E**) Dynamic MCs colonization (% particle colonization). (**F**) MT morphological comparison through SEM imaging of static vs. dynamic MCs colonization. Arrows point at homogenous cell distribution along MTs. Scale bars: Static conditions = 100 µm; dynamic conditions = 400 µm (7), 200 µm (8), 1000 µm (9), 100 µm (10). * *p*-value ≤ 0.05. Arrowheads pointing deposited ECM, ** *p*-value ≤ 0.01, **** *p*-value ≤ 0.0001.

Generally, static seeding is described to be inefficient compared to dynamic seeding procedures, yielding lower efficiency and poor homogeneity [43]. Improved cell-MC attachment using intermittent agitation has been extensively reported against static or continuous regime [44]. To improve cell seeding, MCs colonization was further studied in dynamic conditions using a spinner flask bioreactor [16,45,46,47] to homogenize the cellular distribution and CDM deposition along the MTs. An intermittent stirring regime, serum deprivation, and cell/MCs ratio were investigated as main parameters involved in the optimal MCs colonization. Cell cultures were stirred intermittently at 30 rpm using two different stirring regimes for eight hours—8 h: 3 min 30 rpm/27 min 0 rpm and 15 min 30 rpm/15 min 0 rpm. Up to 94.37% of MCs were colonized after 8-h cell seeding applying a 3-min stirring regime, whereas only 43.29% of MCs were colonized in the 15 min regimen (Figure 3A). These differences in MC colonization could be explained by higher cell death resulting from longer stirring periods, as well as by an increased number of cell clusters. Total deprivation of FBS showed a significant decrease (60.6%) in MCs colonization compared to the FBS supplemented cell media (94.4%) (Figure 3B). Although serum usage in cell seeding is still controversial [48], our results indicate that its presence is critical for cell adhesion to MCs. This increase in cell adhesion can be explained by the adsorption of serum protein to functionalized biomaterial surfaces [49,50], demonstrating serum as an indispensable media component for cell adhesion in MCs.

The impact of cell/MC ratio on MCs colonization efficiency was also evaluated. To ensure complete MCs colonization, cell number must exceed the total amount of MCs [51], as the cell/MC ratio can be affected by cell death and aggregates formation, which leads to a smaller number of available cells to attach in MCs’ surface. On the other hand, using higher cell/bead ratios may result in cellular aggregates, due to cell-cell interactions. The appropriate ratio relies on MC’s size and its surface area. For PLA MCs with an average surface area of 2000 cm^2^/g, cell/MC ratios higher than 10 cells/bead resulted in significantly lower MC colonization and promoted cell-cell aggregates (12 cells/MC 83.2%). Instead, no statistically significant differences were observed between 8.3 (86.3%) and 10 cells/MC (93.6%), choosing this last condition for further experiments (Figure 3C).

The effect of time seeding on the MCs colonization was also assessed. Results showed a progressive decrease in particle colonization at longer seeding times (Figure 3D,E). The maximum microcarrier colonization rate (MCCR) was observed after the first 4 h, where 94.4% of the MCs were colonized. Although non-statistically significant differences were observed, the colonization ratio decreased around 10% after 8 h of intermittent stirring. Finally, long-term cell-seeding in spinner flasks was not able to support cell proliferation in PLA MCs, as seen by the low cell survival rates observed after 24 and 48 h. We hypothesize that rBM-MSCs do not survive for long periods without physical support to attach, and together with prolonged induced agitation-stress, this results in reduced cell survival and MC adhesion [52].

After MC colonization, cell-seeded MCs were transferred into low attachment 24-well plates to allow cell proliferation and ECM deposition (static cell culture), as this culture system displayed improved outcomes. Plates were titled in 45°,45° allowing cell-seeded MCs to sink down to the wells’ bottom. After 21 days of culture, MTs were studied with SEM (Figure 3F). The average size ranged from 4–7 mm length × 1.5–2.5 mm width × 0.5 mm depth. Qualitative differences were observed between statically seeded MCs (Figure 3(F1–F6)) and stirred seeded MCs (Figure 3(F7–F10)). The stirred seeding favored a homogenous cell distribution in MCs, which led to a uniform ECM deposition within MTs’ core (Figure 3(F8)) compared to static seeding where cells and ECM were mostly found on MTs’ surface [53,54]. Therefore, in the following experiments, CDM MTs were produced using 3 min intermittent agitation, with serum-supplemented medium, at 10 cell/MC ratio and a total cell seeding period of 4 h.

### 3.3. Microtissue Production and Characterization Using PLA, Cultispher^®^ S, and Combining Both Microcarriersthe Combination of Both Microcarriers

**Figure 4:**
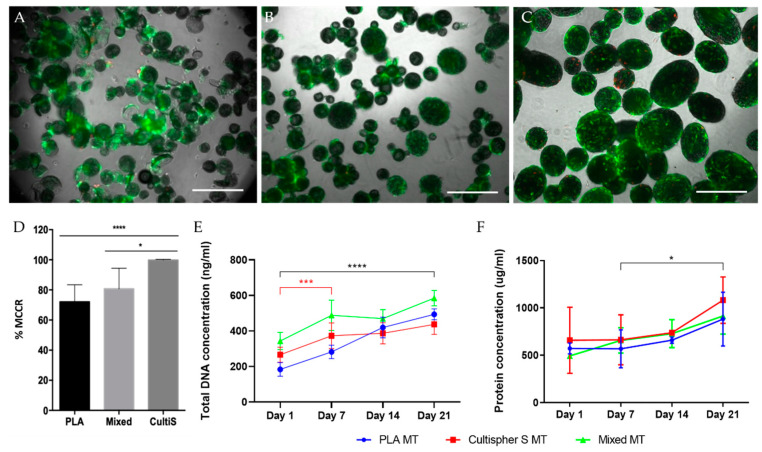
PLA, Cultispher^®^ S and **mixed** MT development. (**A**) Live-dead staining of cell-seeded PLA MCs, scale bar = 500 µm. (**B**) Live-dead staining of cell-seeded mixed MCs, scale bar = 500 µm. (**C**) Live-dead staining of cell-seeded Cultispher^®^ S MCs, scale bar = 500 µm. Alive cells (green), dead cells (red). (**D**) Percentage of particle colonization (MCCR). (**E**) Cell proliferation over MT development. (**F**) Protein deposition over MT development. * *p*-value ≤ 0.05, *** *p*-value ≤ 0.001, **** *p*-value ≤ 0.0001.

In order to evaluate the effect of materials on cell behavior and CDM deposition, PLA MTs’ were studied and compared to MTs produced using the commercially available Cultispher^®^ S MCs and the combination of both MC types (mixed MTs). Initially, MC cell adhesion, survival (Figure 4A–C), proliferation, and protein deposition were assessed for every condition. All MC types successfully allowed cell adhesion, with Cultispher^®^ S MCs (99.8% MCCR) showing the highest cell colonization compared to the other conditions (PLA MTs: 72.0%; mixed MTs: 80.7% MCCR) (Figure 4D). These differences are related to MC composition, porosity, roughness, and size. Gelatin, the main component of Cultispher^®^ S MCs, is a natural polymer that inherently contains important biological cues for protein adsorption and cell adhesion [55].

Cell proliferation was assessed at 1, 7, and 21 days (Figure 4E). The combination of both MC types (mixed MTs) allowed a sustained increased proliferation up to 21 days (total DNA content at Day 1: 388.36 ± 60.34 ng/mL, and at Day 21: 584.99 ± 40.62 ng/mL) compared to the other tested conditions. Cultispher^®^ S MTs showed an increased cellular DNA content from day 1 (266.22 ± 39.91 ng/mL of DNA) to day 7 (372.78 ± 67.48 ng/mL of DNA), but no significant proliferation was observed until the end of the culture (436.80 ± 53.03 ng/mL of DNA), probably due to restricted available surface for cell division [56] leading to a CDM deposition state. Finally, rBM-MSCs seeded on PLA MCs significantly proliferated between day 1 (183.34 ± 36.88 ng/mL of DNA) and 21 (493.50 ± 29.14 ng/mL of DNA), suggesting both polymeric MCs support cell proliferation.

Protein deposition was assessed during the MT formation process (Figure 4F), showing an increase from day 7 until day 21 for all conditions, but not between day 1 and 7. These results correlate with our findings regarding cell proliferation. During the first seven days of culture, cells show a proliferative behavior, whereas from day 7 until day 21, cells enter a protein deposition state. Regarding Cultispher^®^ S and mixed MTs, no significant increase was observed over time within conditions. No statistical differences were observed between conditions.

**Figure 5:**
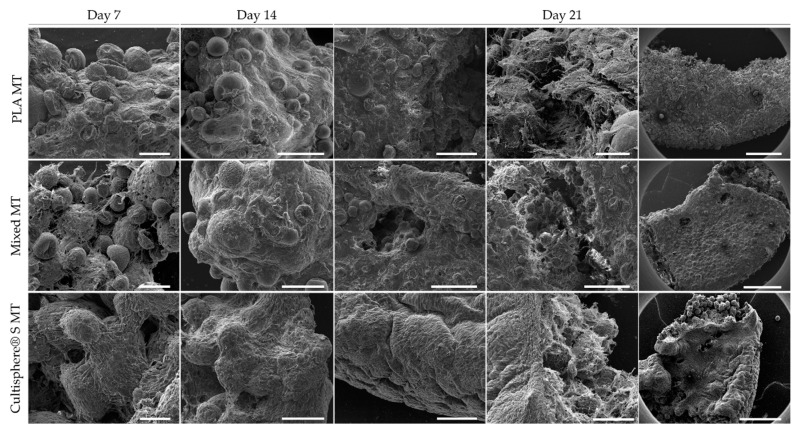
Cell-derived matrix (CDM) deposition assessment through SEM microscopy, scale bars = 100 μm (Day 7), 200 µm (day 14), 50–1000 µm (day 21).

**Figure A2:**
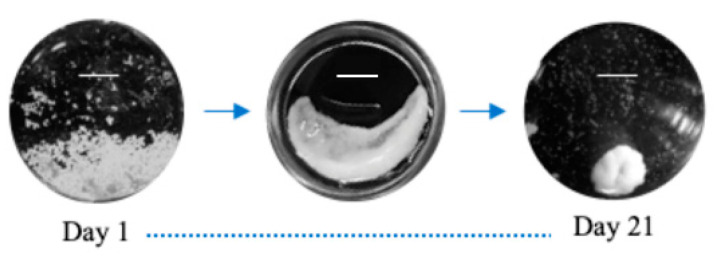
MT contraction process. Scale bars = 2 mm.

After 7, 14, and 21-days culture, all conditions were analyzed by SEM (Figure 5). After seven days, MT structures with evident CDM deposition, were already observed. MCs from all conditions appeared embedded in ECM, highlighting PLA MTs as the condition showing higher CDM density. From day 7, PLA MCs were successfully embedded by CDM, increasing its density over time. SEM images also showed a complete MC embedding in Cultispher^®^ S and mixed MTs after 21-day culture. Cultispher^®^ S and mixed MTs exhibit a denser surface and core deposited CDM, suggesting Cultispher^®^ S MCs role enhancing CDM deposition and the architecture provided by combining the two MC types, resulting in bigger spaces between particles to be filled by cells and deposited CDM. Therefore, Cultispher^®^ S MTs allowed higher CDM density after a 21-day culture period. Another remarkable observation was the contraction process that MTs underwent during the 21-day culture (Figure A2). Over time, cells exert forces and deposit high amounts of CDM that induce tissue contractility, stiffening, and therefore, an increase in protein density.

SEM micrographs (Figure 5), focusing on MTs’ surface, suggested different protein deposition densities and distributions between different MT types. Higher CDM density was observed on Cultispher^®^ S MTs compared to the other MTs. Differences in CDM morphology were also observed between MTs. CDM deposited on Cultispher^®^ S MTs displaying an aligned pattern, while CDM on mixed, and especially on PLA, MTs appeared in a more random distribution. Moreover, some areas on the surface of PLA MTs were not completely covered. However, CDM density in MTs’ core was qualitatively higher in mixed MTs. We hypothesize that higher mixed MTs porosity than in PLA MTs, but smaller than Cultispher^®^ S MTs, together with the combination of both biomaterials, might provide a more suitable microenvironment for cells to deposit ECM proteins successfully embedding all MCs. Cell-seeded MCs successfully aggregated forming MTs constructs on all MCs conditions, mainly attributed to cell-cell interactions and CDM deposition [47].

We hypothesize that the porosity of mixed MTs, together with the combination of both biomaterials, might provide a proper microenvironment that promotes cell ECM deposition.

Together, no differences were observed in cell proliferation and ECM proteins deposition after 21-day culture. Moreover, cell-seeded MCs were successfully embedded in CDM, obtaining robust MTs.

### 3.4. Microtissue Cell-Derived Matrix Proteins Immunofluorescence Staining

**Figure 6:**
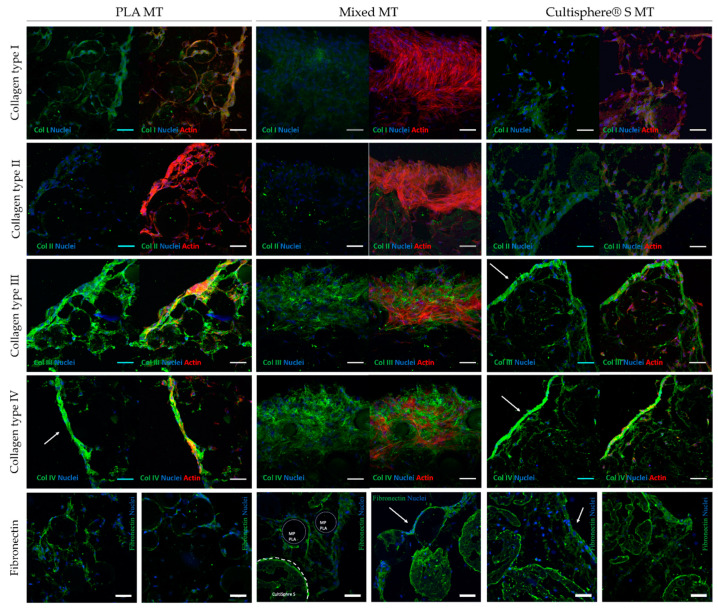
Immunofluorescence staining of sliced MTs for collagen types I, II, III, IV, and fibronectin (green), actin (red), and nuclei (blue), scale bars = 100 µm. The arrowheads point to protein localization.

MTs’ biochemical composition was assessed and compared among conditions. Collagen types I, II, III, and IV expression was analyzed with immunofluorescence staining. All MT conditions deposited all collagen types and fibronectin, although Collagen type IV was qualitatively the most abundant of all analyzed proteins for all three MT conditions (Figure 6) in line with Marinkovic et al. findings [57]. In PLA and Cultispher^®^ S MTs, collagen type IV was localized mostly at their surface, resembling the basement membrane structure [58,59]. Instead, this protein was distributed more homogenously in mixed MTs. Collagen type III was also deposited in all MC types. Differences in protein distribution were observed between collagen type IV and collagen III, being the least present throughout the whole MT structure. This result suggests the presence of a more fibrous MT core. Low deposition of collagen I and especially collagen II was observed in all MTs with no qualitative differences between conditions (Figure 6). As collagen type I was under expressed compared to collagen types III and IV we hypothesize that PLA MC collagen type I functionalization might hinder this protein expression, as cells can sense it in MCs’ surface. Regarding collagen type II, limited deposition of this protein has been reported in chondrogenic factors-free culture medium [60,61,62]. Moreover, MC composition or mechanical properties can also affect BM-MSCs protein expression [63].

Figure 6 also shows fibronectin surrounding Cultispher^®^ S MCs in small amounts, both in mixed and Cultispher^®^ S MTs. Instead, fibronectin found in PLA MTs was qualitatively higher in abundance than in the other two conditions. This protein was found both in the surface and core of PLA MTs, although it seemed to be strongly localized on MT’s surface. We hypothesize that size and space between MCs can play an important role in MC interconnectivity by CDM deposition. Higher particle size and distance between particles can hinder cells from properly interconnecting MCs, and therefore, this can affect MT structural integrity. Finally, MC composition can also have an impact on the type and amount of deposited CDM. Nevertheless, all MT conditions showed good integrity when manipulated, due to the total amount of deposited CDM.

### 3.5. Microtissues Angiogenesis Ex Vivo

**Figure 7:**
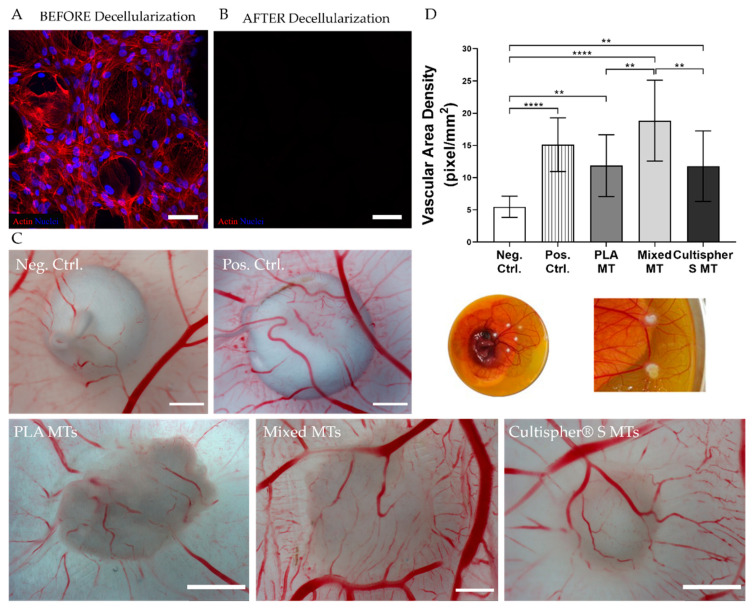
Ex vivo MTs angiogenesis potential. (**A**) MT staining before decellularization and (**B**) after decellularization. Actin (red), nuclei (blue), scale bars = 100 µm. (**C**) Ex vivo stereomicroscope images from tested conditions, scale bars = 2 mm. (**D**) Vascular area density quantification. ** *p*-value ≤ 0.01, **** *p*-value ≤ 0.0001.

Since the angiogenic response of most injured tissues is crucial for their regeneration, the angiogenic potential of our developed MTs was evaluated using the CAM ex vivo model, which serves as an indicator for pro- or antiangiogenic potential of tested biomaterials or substances [34,64]. Before MT implantation into chick embryos, a decellularization process was performed to remove rBM-MSCs. Decellularized MTs did not show any cellular nuclei, suggesting effective decellularization for all conditions (Figure 7A,B).

Chick embryos were placed in Petri dishes and implanted with decellularized MTs. PCL polymeric macroparticles scaffolds matching MTs size loaded with VEGF (positive control) or alone (negative control) were used as controls.

MTs were found to be fully integrated within the CAM, and vessel formation was clearly visible (Figure 7C). A significant increase in vessel density was observed in all MTs compared to the negative control, suggesting a vascularization-induction role from deposited CDM in MTs. Interestingly, these levels were higher in mixed MTs (18.86 ± 6.27 pixels/mm^2^) compared to the other MTs (PLA MTs 11.43 ± 4.73 pixels/mm^2^; Cultispher^®^ S MTs 10.83 ± 4.06 pixels/mm^2^) or negative PCL controls (5.47 ± 1.64 pixels/mm^2^) and similar compared to VEGF-loaded PCL scaffolds. (14.75 ± 4.74 pixels/mm^2^, Figure 7D).

It has been shown that ECM proteins, as well as pro-angiogenic factors, retained in the ECM after the decellularization process, promote the formation of blood vessels [65]. The higher angiogenic response observed in mixed MTs may be explained by the amount of collagen produced, the MC-specific MT architecture, and the specific ECM organization [66]. Upholding our observations, researchers have described changes in newly generated microvessels size depending on the amount and density of collagen from the ECM [67]. Indeed, abundant interstitial collagen fibers raised more functional and mature capillaries [68]. In addition, higher MC porosity, as well as reduced density in ECM found in mixed MTs, might provide a suitable microenvironment for endothelial cells to migrate within MTs and generate a complex vasculature network providing the necessary nutrients and oxygen for tissue repair and regeneration.

Altogether, these results highlight a great potential for mixed MC MTs in tissue neovascularization to provide nutrients and oxygen during tissue repair and regeneration processes. As observed, distinct MC material, size, and mechanical properties can greatly influence the properties of the deposited CDM, and subsequently, their potential in different tissue engineering applications. Moreover, MC-free CDM MTs can serve as platforms to better mimic and study tissue microenvironments for regenerative purposes or to study disease models.

## 4. Conclusions

In this work, we have defined a strategy for the formation of CDM MTs using rBM-MSCs. MC culture and MT formation are promising strategies to generate CDM scaffolds for modular TE approaches. The deposited CDM may greatly influence cell behavior from surrounding tissues and induce spontaneous and effective tissue regeneration mimicking the native tissue structure, composition, and mechanical properties. Therefore, our findings suggest that the nature of the biomaterial used, its architecture, and the cell source greatly impact MT features. Moreover, tuning up these parameters allows researchers to develop tailor-made CDM according to the tissue regeneration/disease modeling objective. We observed that all MT conditions generate a dense ECM embedding MCs without using growth factors. Interestingly, by mixing both types of MCs, mixed MTs, we were able to observe continuous cell proliferation, secretion of an abundant ECM, and promotion of angiogenic response, a crucial step for bone, skin, or muscle tissue regeneration, as well as to study cancer disease progression and metastasis. Together, these results reinforce the need to study the interactions between biomaterials—and in particular, MCs templates—and cells to develop 3D structures that mimic specific tissue microenvironments, with the purpose of studying tissue regeneration and disease progression processes.

## Figures and Tables

**Table 1:** Antibodies used in immunofluorescent staining.

Primary Antibodies			Secondary Antibodies			
Reactivity	Host	Ref.	Reactivity	Host	Emission	Ref.
Collagen type I	Mouse	ab6308	Mouse	Goat	488 nm	ab150117
Collagen type II	Rabbit	ab34712	Rabbit	Goat	488 nm	ab150081
Collagen type III	Rabbit	ab7778	Mouse	Donkey	594 nm	SAB4600098
Collagen type IV	Rabbit	ab6586				
Fibronectin	Rabbit	ab2413				
Actin	Mouse	sc-47778				
Laminin	Rabbit	ab11575				

## Data Availability

The data presented in this study are available upon request from the corresponding author.

## Supplementary Materials

The following are available online at https://www.mdpi.com/2227-9059/9/3/232/s1, Fiji Macro S1: CAM_quantification_macro.ijm.
