# Supplementary material for: Development and Angiogenic Potential of Cell-Derived Microtissues Using Microcarrier-Template
Source: Biomedicines. 2021 Feb 25;9(3):232. doi: 10.3390/biomedicines9030232 (PMC8025087; doi:10.3390/biomedicines9030232)
Supplement: Supplementary file 1 [file biomedicines-09-00232-s001.zip › Supplementary Figures/Supplementary Figures captions.docx]

**Figure S1. MT SEM images** from A-C) U96 well-plates with 25,000 cells/mg MC, 12,500 cells/mg MC two-step seeding and 50,000 cells/mg MC, scale bars = 200 µm; D-F) 24 well-plates with 25,000 cells/mg MC, 12,500 cells/mg MC two-step seeding and 50,000 cells/mg MC, scale bars = 500 µm; and G-I) transversal sections from 24 well-plates MTs with 25,000 cells/mg MC, 12,500 cells/mg MC two-step seeding and 50,000 cells/mg MC, scale bars = 100 µm. Arrowheads pointing fibrillar ECM.

**Figure S2. MT contraction process.** Scale bars = 2mm.
