# Supplementary figures and images for: Development and Angiogenic Potential of Cell-Derived Microtissues Using Microcarrier-Template
Source: Biomedicines. 2021 Feb 25;9(3):232. doi: 10.3390/biomedicines9030232 (PMC8025087; doi:10.3390/biomedicines9030232)

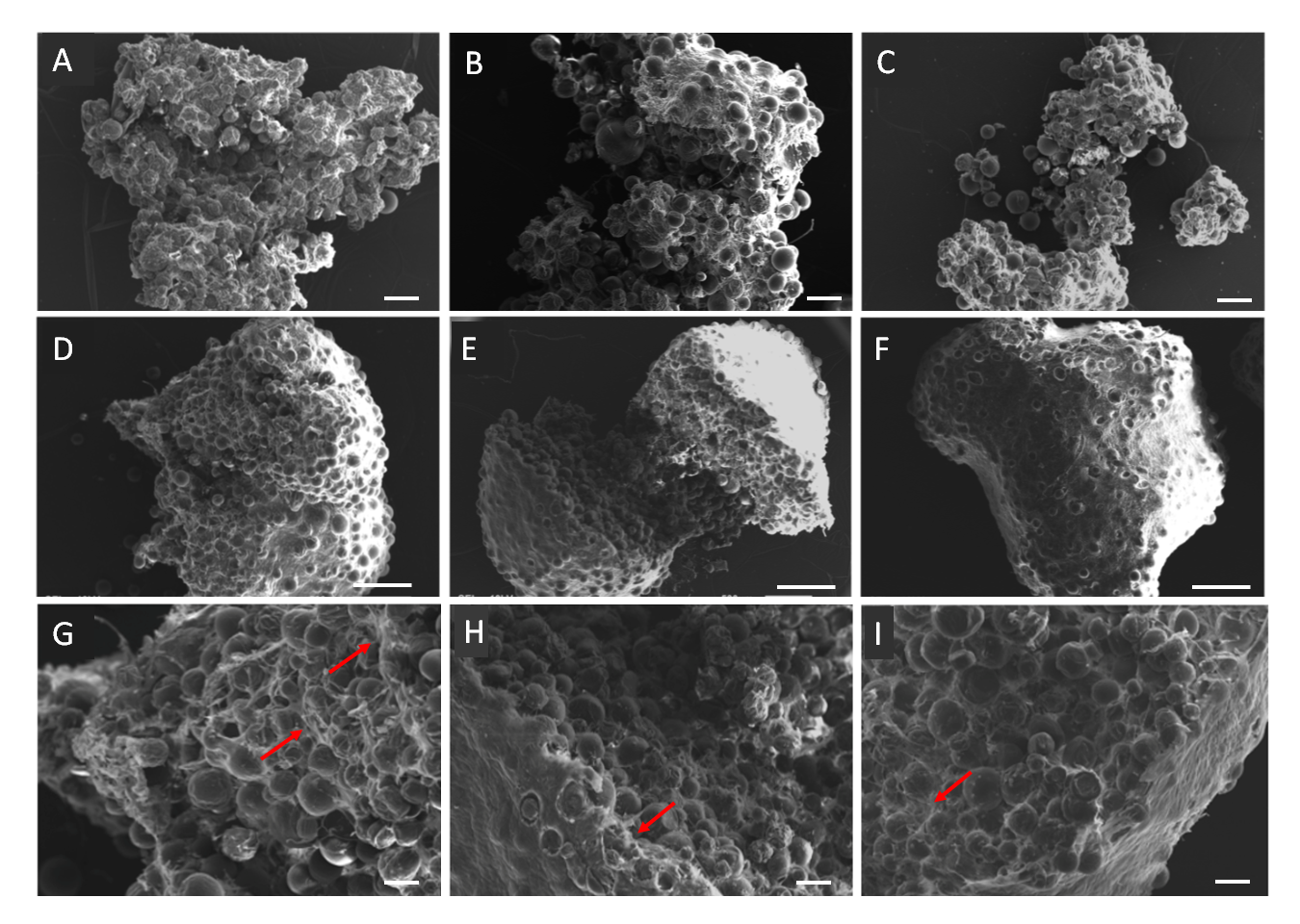

Supplement: Supplementary file 1 [file biomedicines-09-00232-s001.zip › Supplementary Figures/Supplementary figure 1.tif]

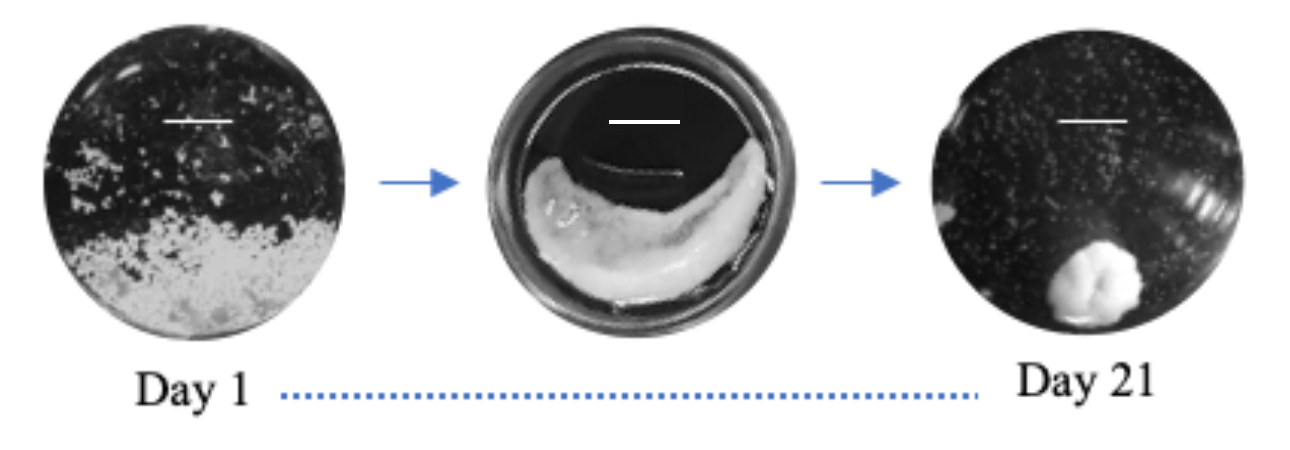

Supplement: Supplementary file 1 [file biomedicines-09-00232-s001.zip › Supplementary Figures/Supplementary figure 2.tif]
